# Dynamics of axonal β‐actin mRNA in live hippocampal neurons

**DOI:** 10.1111/tra.12865

**Published:** 2022-08-31

**Authors:** Byung Hun Lee, Seokyoung Bang, Seung‐Ryeol Lee, Noo Li Jeon, Hye Yoon Park

**Affiliations:** ^1^ Department of Physics and Astronomy Seoul National University Seoul Republic of Korea; ^2^ Department of Mechanical Engineering Seoul National University Seoul Republic of Korea; ^3^ Department of Medical Biotechnology Dongguk University Goyang Republic of Korea; ^4^ The Institute of Applied Physics Seoul National University Seoul Republic of Korea; ^5^ Department of Electrical and Computer Engineering University of Minnesota Minneapolis Minnesota USA

**Keywords:** actin patch, axonal β‐actin mRNA, live‐cell imaging, microfluidic device, mRNA localization process

## Abstract

Localization of mRNA facilitates spatiotemporally controlled protein expression in neurons. In axons, mRNA transport followed by local protein synthesis plays a critical role in axonal growth and guidance. However, it is not yet clearly understood how mRNA is transported to axonal subcellular sites and what regulates axonal mRNA localization. Using a transgenic mouse model in which endogenous β‐actin mRNA is fluorescently labeled, we investigated β‐actin mRNA movement in axons of hippocampal neurons. We cultured neurons in microfluidic devices to separate axons from dendrites and performed single‐particle tracking of axonal β‐actin mRNA. Compared with dendritic β‐actin mRNA, axonal β‐actin mRNA showed less directed motion and exhibited mostly subdiffusive motion, especially near filopodia and boutons in mature dissociated hippocampal neurons. We found that axonal β‐actin mRNA was likely to colocalize with actin patches (APs), regions that have a high density of filamentous actin (F‐actin) and are known to have a role in branch initiation. Moreover, simultaneous imaging of F‐actin and axonal β‐actin mRNA in live neurons revealed that moving β‐actin mRNA tended to be docked in the APs. Our findings reveal that axonal β‐actin mRNA localization is facilitated by actin networks and suggest that localized β‐actin mRNA plays a potential role in axon branch formation.

## Graphical abstract

In this study, we imaged β‐actin mRNA in live axons of hippocampal neurons. Compared with β‐actin mRNA in dendrites, axonal β‐actin mRNAs exhibited less directed motion with slower speed. The majority of them were localized to actin patches (APs) which are precursors of the axonal branch and showed confined diffusive motion within the APs.

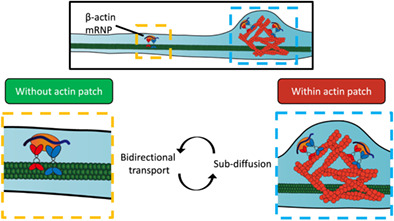

## 1 INTRODUCTION

Nonuniform localization of mRNA is important for efficient protein supply to the various subcellular compartments.^1^ In neurons, spatiotemporally regulated local protein synthesis following mRNA localization provides proteins for neuronal development, plasticity, and survival.^2^ mRNAs are recruited to messenger ribonucleoprotein (mRNP) complexes and subsequently transported to dendrites and axons by multiple motor proteins.^1^, ^3^ For example, β‐actin mRNA contains a *cis*‐acting element in its 3′‐untranslated region (UTR) that is recognized by zipcode‐binding protein 1 (ZBP1, the homolog of Vg1RBP and IMP‐1). The β‐actin mRNA‐ZBP1 complex is transported to dendritic spines,^4^ axonal growth cones,^5^ and axonal branch points^6^ by motor proteins such as kinesin,^7^, ^8^ dynein,^8^ and myosin‐Va.^9^

In axons, which project a long distance from the soma, mRNA localization in subcompartments such as protrusions, branch points, and growth cones is important for axonal navigation, branching, and maintenance.^10^ Because local translation is a critical factor for the local proteome in neurites, an appropriate transport mechanism is required for mRNA to reach the target site; this transport process has been implicated as important in a number of neurological disorders.^11^, ^12^ Recent advances in mRNA labeling techniques have enabled single mRNA imaging in live neurons^13^; thus, real‐time visualization of mRNA localization process has become possible. Tracking the movement of specific mRNA can help decipher how mRNA localization patterns are generated. In dendrites, β‐actin mRNA has been reported to show motor‐driven transport and follow an aging Lévy walk, which is an efficient random walk strategy to find randomly distributed targets.^14^ In addition, a recent study performed single‐molecule imaging of β‐actin mRNA in live axons and demonstrated how anterograde/retrograde bias of mRNA movement led to the localization of β‐actin mRNA in axonal growth cones.^5^ To our knowledge, however, the process by which axonal β‐actin mRNA is localized to potential branch points is still unknown, although it was shown that local translation of axonal β‐actin mRNA is required for branch emergence.^15^ Moreover, most studies on axonal mRNA trafficking have been performed using sensory neurons^6^ or retinal neurons of *Xenopus*.^5^, ^15^, ^16^, ^17^, ^18^ However, the transport and localization of axonal mRNA compared with dendritic mRNA have not been studied in other neuronal cell types such as hippocampal neurons.

Here, we investigated the dynamics and movement of single β‐actin mRNA particles in the axons of dissociated hippocampal neurons. To visualize single β‐actin mRNA in live neurons, we used a genetically engineered mouse model called the MCP × MBS mouse, in which every endogenous β‐actin mRNA is labeled with up to 48 green fluorescent protein (GFP) molecules.^19^ We compared the movement of β‐actin mRNA in axons and dendrites, finding that axonal β‐actin mRNA showed less directed motion and slower speed than dendritic mRNA. Furthermore, ~40% of axonal β‐actin mRNA was localized in filopodia and boutons and showed confined diffusion. Because filopodia and boutons are actin‐rich areas,^20^ we hypothesized that the localization of β‐actin mRNA in axons is facilitated by actin patches (APs), which are regions with a high density of filamentous actin (F‐actin). By observing both mRNA and APs, we found that axonal β‐actin mRNA was localized mostly in APs and showed confined diffusion within these regions. Our report describes the real‐time observation of axonal β‐actin mRNA localization into branch points and how APs mediate mRNA localization.

## 2 RESULTS

### 2.1 Axonal and dendritic β‐actin mRNAs exhibit different types of motion

**FIGURE 1:**
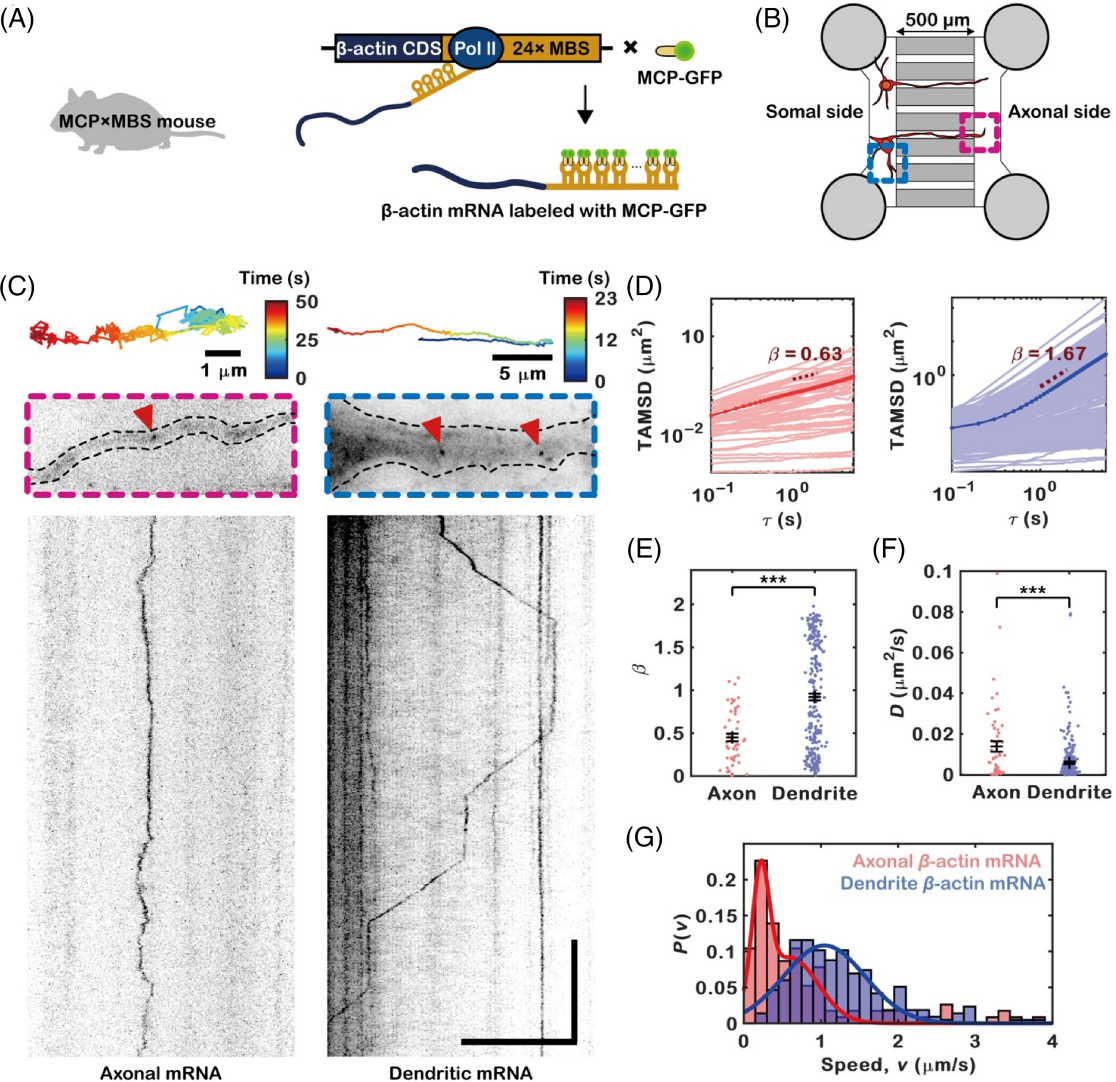
Axonal and dendritic β‐actin mRNA molecules exhibit different motion patterns. (A) Schematic for labeling β‐actin mRNA with GFP molecules. MCP binds to the MBS RNA stem loop with high specificity and affinity. The MCP × MBS mouse expresses endogenous β‐actin mRNA labeled with up to 48 green fluorescent protein molecules. (B) Schematic diagram of the microfluidic device, consisting of four reservoirs (gray circles) and dozens of channels (500‐μm long, 10‐μm wide). Neurons were cultured on one side of the chamber, and axons grew within the channels and into the other side of the chamber. Dendritic β‐actin mRNAs were observed from the side that was seeded with neurons (blue box), and axonal β‐actin mRNAs were observed from the opposite side (pink box). (C) Top, representative axonal and dendritic β‐actin mRNA molecule tracks colored according to time. Middle, snapshot of time‐lapse images of β‐actin mRNA molecules in an axon (left) and a dendrite (right). Red arrows indicate the β‐actin mRNA molecules. Bottom, kymographs of the time‐lapse images. The horizontal and vertical axes show the position and time, respectively. Scale bars = 10 μm (horizontal) and 10 s (vertical). (D) Time‐averaged mean square displacements (TAMSDs) were calculated from axonal (left, *n* = 53 mRNA molecules) and dendritic (right, *n* = 215 mRNA molecules) mRNA tracks. The thick lines indicate the average TAMSD values, and the dashed lines indicate the linearly fitted region. (E) TAMSD exponents of axonal and dendritic mRNA molecules (***p < 10^−10^ by the two‐sample Kolmogorov–Smirnov test). (F) Diffusion coefficients of axonal and dendritic mRNA molecules (*** *p* < 0.001 by the two‐sample Kolmogorov–Smirnov test). (G) Probability distribution of the speed of axonal (red) and dendritic mRNAs (blue). The histograms were fitted with a bimodal distribution (red) and a Gaussian distribution (blue) for axonal and dendritic β‐actin mRNA, respectively. The error bars represent the standard error of the mean (SEM).

Previous methods to study mRNA transport include imaging fluorescently labeled RNA‐binding proteins (RBPs)^9^, ^17^ or nonspecifically labeled RNAs.^15^ However, these methods cannot visualize the movement of particular species of endogenous mRNA. To visualize endogenous β‐actin mRNA, we used MCP × MBS mice, a previously developed mouse model that not only expresses GFP‐fused MS2 bacteriophage capsid protein (MCP‐GFP) but also harbors an insert of 24 repeats of the MS2‐binding site (MBS) in the 3′‐UTR of the β‐actin gene (Figure 1A). Because MCP‐GFP dimerizes and specifically binds to an MBS RNA stem loop, each β‐actin mRNA can be labeled with up to 48 GFP molecules (24 stem loops × 2) and can be visualized as a single particle using highly inclined and laminated optical sheet (HILO) microscopy.^21^ To separate axons from other neuronal compartments, we cultured hippocampal neurons from MCP × MBS mice on a microfluidic device consisting of two regions connected by several narrow channels (500‐μm long, 10‐μm wide) in the middle (Figure 1B).^22^ We seeded neurons on one side of the microfluidic device, and the channels acted as a filter to isolate axons from cell bodies and dendrites, allowing us to observe axonal β‐actin mRNA on the other side.

We found several qualitative differences in the movement of β‐actin mRNA in axons and dendrites. While β‐actin mRNA in dendrites frequently showed directed transport (Figure 1C, right panel, Movie S1), most axonal β‐actin mRNA exhibited confined diffusive motion (Figure 1C, left panel, Movie S2). To characterize the movement of β‐actin mRNA quantitatively, we recorded the trajectories of β‐actin mRNAs by using HybTrack software, a type of tracking software that combines manual and automatic detection.^23^ We calculated the time‐averaged mean square displacement (TAMSD) of the β‐actin mRNA tracks (Figure 1D). The mean square displacement (MSD) of a diffusing molecule can be written as ~*τ* ^*β*^, and the type of diffusion can be roughly classified according to the value of *β* (0 < *β* < 1: subdiffusion; *β* > 1: superdiffusion), which can be obtained by linear fitting on a log–log scale.^24^, ^25^ While a number of dendritic β‐actin mRNAs showed superdiffusive motion as a result of directed transport, most of the axonal β‐actin mRNA exhibited subdiffusive motion (Figure 1D,E). However, the local diffusion coefficients of axonal β‐actin mRNAs were higher than those of dendritic β‐actin mRNAs (Figure 1F).

Because the mRNA movement could differ depending on the proximity to the soma, we also compared the movement of β‐actin mRNA in proximal (<100 μm from soma) and distal (>100 μm from soma) dendrites. We found that the fraction of β‐actin mRNA showing directed movement was smaller in distal dendrites (6.5% during 1‐min imaging time) than in proximal dendrites (21% during 1‐min imaging time). And β‐actin mRNAs in distal dendrites had lower MSD exponents and diffusion coefficients than those in proximal dendrites (Figure S1). Although β‐actin mRNAs in distal dendrites showed different mobility from those in proximal dendrites, they were both distinct from axonal β‐actin mRNAs. While it was very hard to find any directed motion of β‐actin mRNA in axons during 1‐min imaging time, we were able to detect a few mRNAs showing directed motion in distal dendrites. Moreover, β‐actin mRNAs in both proximal and distal dendrites had much lower diffusion coefficients than axonal β‐actin mRNAs. Therefore, there appears to be a fundamental difference between axonal and dendritic mRNA transport in mature hippocampal neurons.

We next measured the movement speed of each β‐actin mRNA molecule along the path in which the molecule moved more than 1.5 μm in the same direction, which was considered to indicate motor‐driven movement. Dendritic β‐actin mRNAs showed higher speed (1.04 ± 0.53 μm/s, fitted with a Gaussian distribution) than axonal β‐actin mRNA (0.22 ± 0.11 and 0.6 ± 0.36 μm/s, fitted with two Gaussian distributions) (Figure 1G). Taken together, these results indicate that axonal β‐actin mRNAs have slower and less directed but more diffusive movement than dendritic mRNAs, suggesting that β‐actin mRNA movement in axons and dendrites is facilitated by different transport mechanisms.

### 2.2 Types of axonal β‐actin mRNA motion in boutons, filopodia, and axon shaft

**FIGURE 2:**
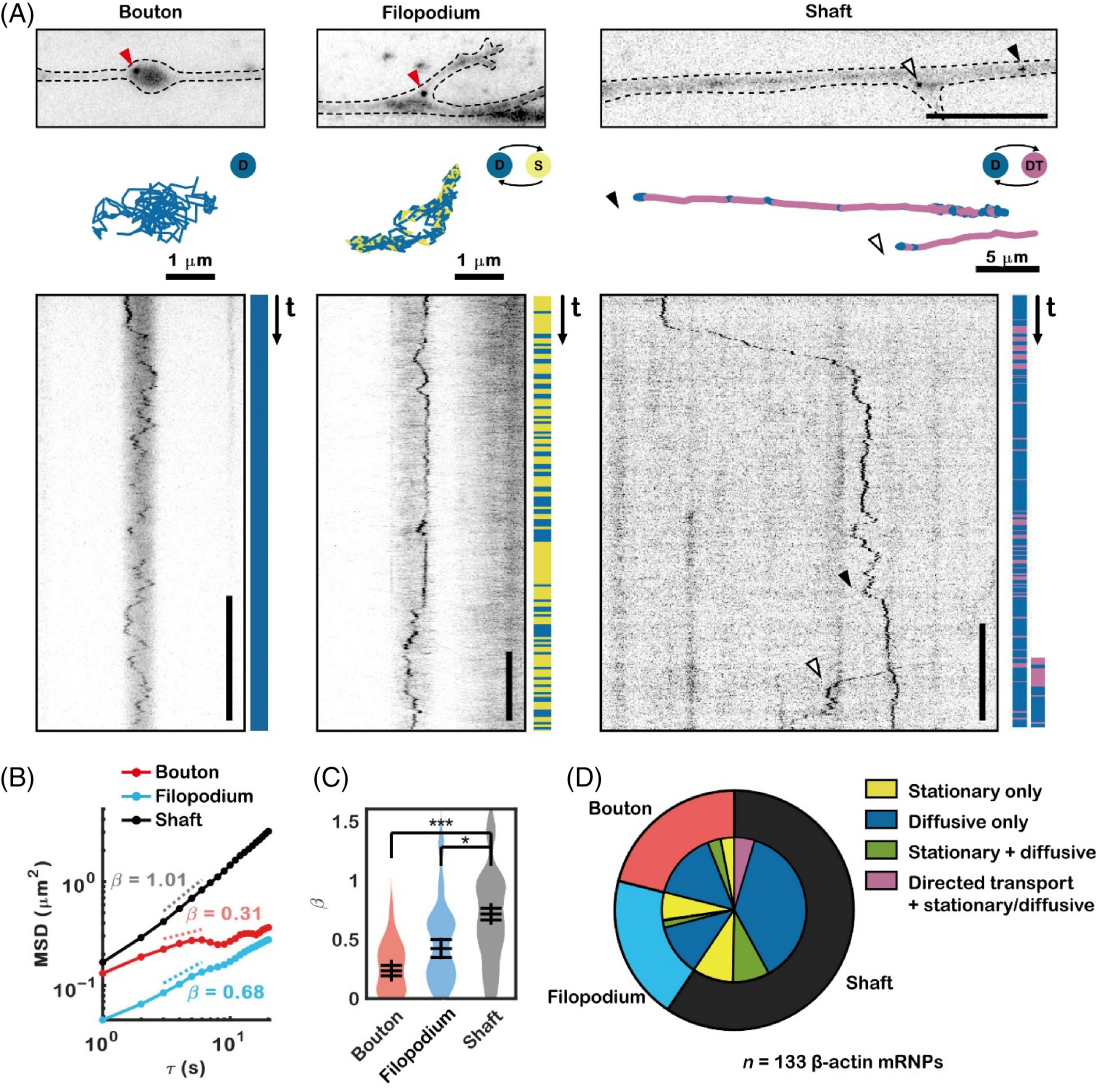
Localization and dynamics of axonal β‐actin mRNA in boutons, filopodia, and shafts. (A) Representative image of axonal β‐actin mRNA in a bouton (left), a filopodium (middle), and a shaft (right). The movement of mRNA molecules was characterized with HMM‐Bayes software, and tracks were colored according to their inferred motion types. S, stationary; D, diffusive; DT, directed transport. Scale bars = 10 μm (horizontal), 10 s (vertical, left, and middle), 5 min (vertical, right). (B) Average time‐averaged mean square displacements (TAMSDs) of tracks of mRNA molecules in boutons, filopodia, and shafts. (C) The exponent values of the TAMSDs of β‐actin mRNAs in boutons, filopodia, and shafts (***p < 10^−4^; *p < 0.05 by the two‐sample Kolmogorov–Smirnov test). The error bars represent the SEM. (D) Pie chart showing the proportions of β‐actin mRNA molecules localized in each subcompartment and the proportions of motion types among them.

We next examined whether the motion of β‐actin mRNA varies among axonal subcompartments. The mRNAs were classified according to their subcompartment locations: boutons, filopodia, and shaft (Figure 2A). We found that some axonal β‐actin mRNA molecules in boutons or near filopodia showed subdiffusive motion (Figure 2A, left and middle panel, Movies S3 and S4). Once the β‐actin mRNAs localized into filopodia or boutons, they rarely exited those subcompartments. Occasionally, some mRNAs shifted from directed transport to diffusive motion near the base of potential filopodium (Figure 2A, right panel, Movie S5). The filopodium elongated a few minutes after the mRNA arrived at that location (Figure S2). We calculated the MSD of the mRNA molecules in each subcompartment (Figure 2B) and found that mRNA molecules in the axon shaft had higher MSD exponent values than those in boutons or filopodia (Figure 2C), which indicates that some mRNA in the shaft was still being directly transported and not yet localized. To identify the types of transient motion undergone by mRNA molecules in each subcompartment, we used HMM‐Bayes software^26^ and classified trajectories into stationary, diffusive, and directed transport motion. Though most mRNA molecules in all three subcompartments showed stationary or diffusive motion, 7.7% of the β‐actin mRNAs in the shaft showed the movement containing directed transport motion, which is characteristic of kinesin/dynein‐mediated microtubule‐dependent transport, during 1–15‐min imaging time window (Figure 2D). Collectively, these data indicate that axonal β‐actin mRNAs occasionally travel a long distance via directed transport in the shaft and dock at a bouton or filopodium for potential local translation.

### 2.3 Colocalization of β‐actin mRNA with APs

**FIGURE 3:**
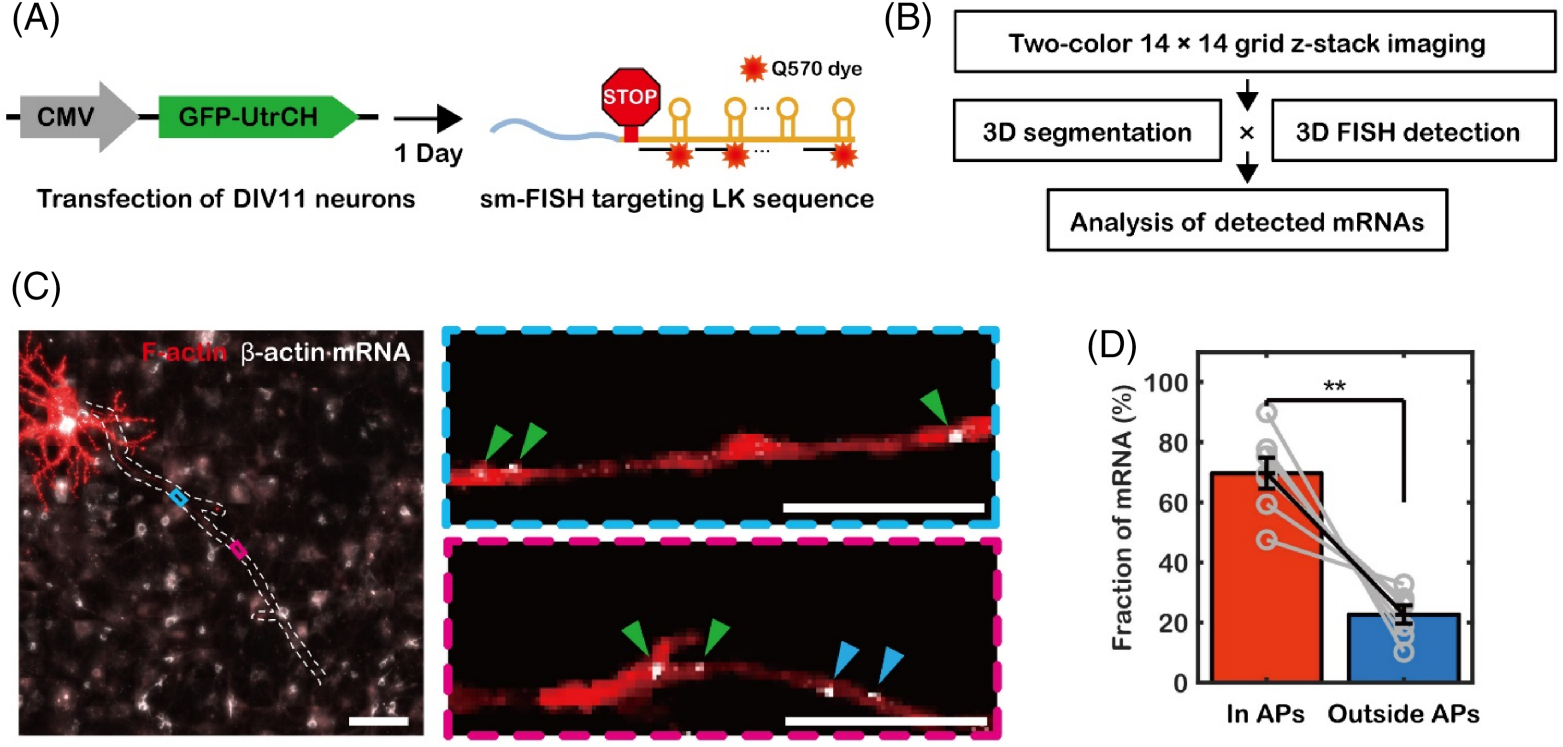
Single‐molecule fluorescence in situ hybridization (smFISH) targeting β‐actin mRNA in sparsely transfected neurons. (A) DIV11 neurons were transfected with CMV‐GFP‐UtrCH to label filamentous actin, and smFISH was performed on the following day. (B) To image axons over a broad area, grid imaging was performed prior to the stitching process. Axonal β‐actin mRNA molecules of the transfected neuron were identified by three‐dimensional (3D) axon segmentation and 3D particle detection. (C) Left, representative smFISH image. The dotted lines delineate an axon. Right, enlarged images of the areas within the blue and pink boxes in the left panel. The green arrows indicate β‐actin mRNA molecules within actin patches (APs), and the blue arrows indicate β‐actin mRNA molecules outside APs. Scale bars = 50 μm (Left panel), 5 μm (right panel). (D) Fractions of β‐actin mRNA molecules colocalized with APs (*n* = 7 axons, 367 mRNAs, **p < 0.01 by pairwise *t*‐test). The error bars represent the SEM.

Although some axonal β‐actin mRNA molecules in the shaft showed directed transport motion, most showed stationary or diffusive motion. The sequence of β‐actin mRNA is known to have a *cis*‐acting element that interacts with ZBP1, an RBP, and the ZBP1 protein associates with myosin‐Va, an actin‐based motor protein in axons.^9^ Moreover, dendritic β‐actin mRNAs have the same cis‐acting element recognized by ZBP1 and are localized to dendritic spines by the F‐actin network.^4^ Therefore, we presumed that the localization of axonal β‐actin mRNA is also facilitated by the F‐actin network. To simultaneously visualize β‐actin mRNA and F‐actin network, we transfected neurons at days in vitro (DIV) 11 with GFP‐fused utrophin calponin homology domain (GFP‐UtrCH), which binds to F‐actin and allows it to be visualized. After transfection, we performed single‐molecule fluorescence in situ hybridization (smFISH) targeting the MBS linker sequence of β‐actin mRNA (Figure 3A). Only a few transfected neurons (~20 neurons per dish) expressed GFP‐UtrCH, which allowed us to identify the axons of individual cells by their morphology. To observe β‐actin mRNA in axons across a large area, we performed grid imaging with a high‐magnification objective lens. Because the smFISH probes targeted β‐actin mRNAs of all neurons in the dish, we detected β‐actin mRNA signal by three‐dimensional particle detection, segmented the axons of transfected neurons, and then identified β‐actin mRNA only in those segmented axons (Figure 3B). Along each axon, we were able to find APs where the F‐actin signal was distinctly bright (Figure 3C), representing precursors of branches.^27^ Because UtrCH expression level was different for each neuron and dependent on the distance from the soma, we segmented AP regions by manual thresholding. Analyzing 364 β‐actin mRNA particles from 7 axons, we found that 70 ± 5% of the mRNAs were colocalized with APs (Figure 3D).

### 2.4 Movement of β‐actin mRNA within APs

**FIGURE 4:**
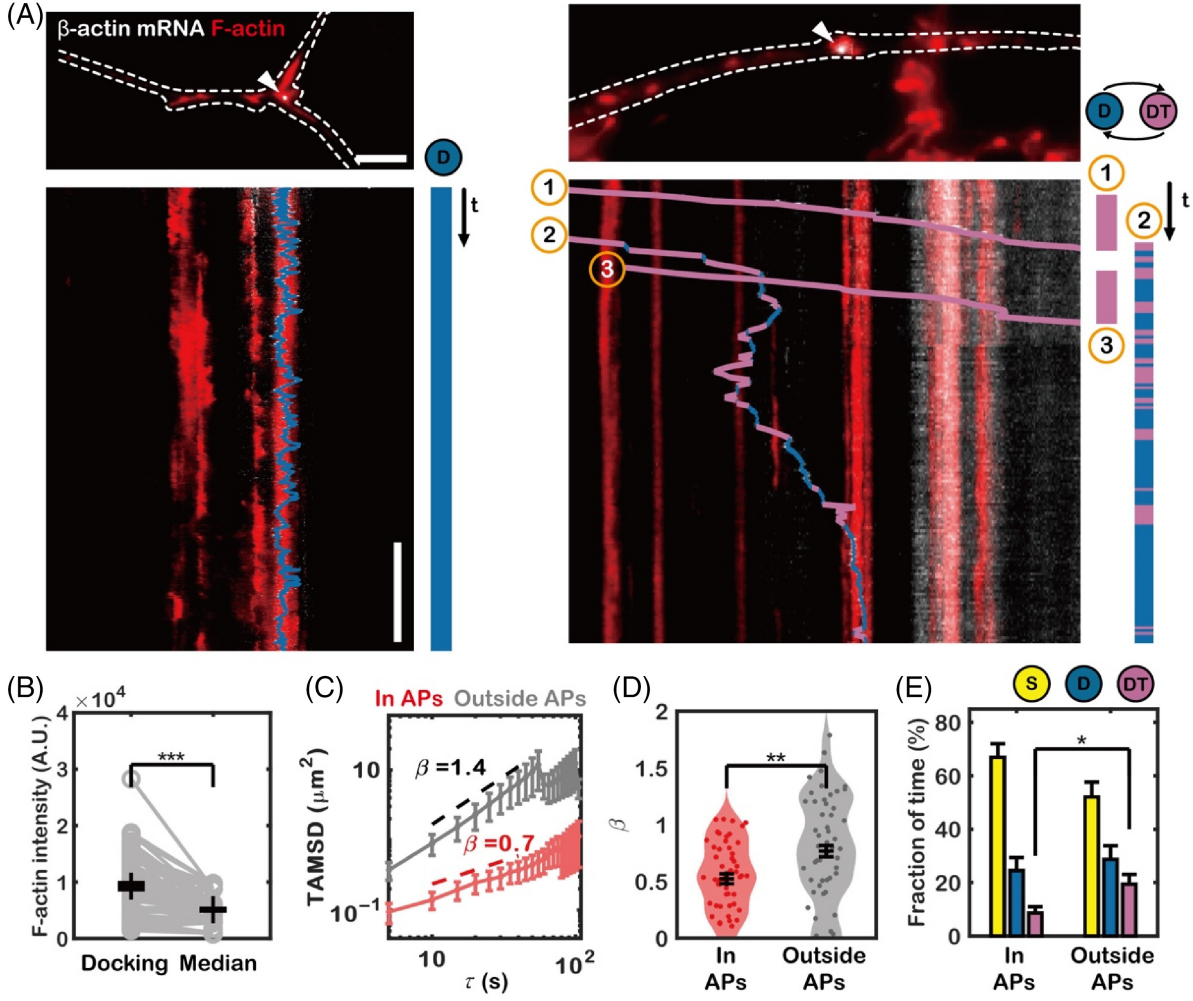
Dual‐color imaging of filamentous actin (F‐actin) and β‐actin mRNA in live neurons. (A) Left, representative image of an axonal β‐actin mRNA localized in an actin patch (AP) and a kymograph of the time‐lapse image. HMM‐Bayes software was used to classify the type of motion exhibited by the mRNA molecule. Right, three β‐actin mRNA molecules traversing an axon. β‐Actin mRNA molecules #1 and #3 showed pure transport motion without docking. However, mRNA molecule #2 showed mixed diffusive and transport motion and docked at an AP. Scale bars = 5 μm (horizontal), 10 min (vertical). (B) The F‐actin intensity at the β‐actin mRNA‐docking site and the median F‐actin intensity in the axon are shown (*n* = 78 mRNAs, ***p < 10^−12^ by pairwise *t*‐test). (C) Time‐averaged mean square displacement (TAMSD) of tracks of β‐actin mRNA inside (red) and outside (gray) APs. (D) Distribution of the TAMSD slope value, β, calculated from the tracks of mRNA molecules colocalized with APs and not colocalized with APs (**p < 0.01 by the two‐sample Kolmogorov–Smirnov test). (E) Fractions of time that β‐actin mRNA exhibited stationary, diffusive, and transport motion (*p < 0.05 by the two‐sample Kolmogorov–Smirnov test). The error bars represent the SEM.

To observe how axonal β‐actin mRNA localizes to APs in real‐time, we performed simultaneous imaging of β‐actin mRNA and APs. To morphologically identify axons, we sparsely transfected MCP × MBS mouse neurons with mCherry‐fused UtrCH and simultaneously imaged F‐actin and β‐actin mRNA over ~30 min using a HILO microscope equipped with two electron‐multiplying charge‐coupled device (EMCCD) cameras. We found that the majority of axonal β‐actin mRNAs were trapped in APs as shown in Figure 4A (left) and Movie S6. Some axonal β‐actin mRNAs simply passed the APs without anchoring (Figure 4A, right, mRNA #1 and #3; Movie S7), but we also observed mRNAs that were anchored to the APs and eventually localized (Figure 4A, right, mRNA #2; Movie S7). Using the HMM‐Bayes approach, we inferred the transient motion types of β‐actin mRNAs, and we interpreted a change in mRNA motion from directed transport to a diffusive or stationary state as “docking.” We found that β‐actin mRNA molecules mostly docked in locations where the F‐actin signal was higher than the median F‐actin signal in the axons (Figure 4B). We then segmented APs by thresholding F‐actin images and analyzed the paths of β‐actin mRNAs inside and outside APs. The β‐actin mRNAs outside APs showed a steeper log‐scaled TAMSD (Figure 4C) and had higher exponent values (Figure 4D) than those inside APs. This result indicates that the β‐actin mRNAs within APs showed directed motion to a lesser extent than β‐actin mRNAs outside APs. Moreover, the HMM‐Bayes analysis indicated that the axonal β‐actin mRNAs had an increased likelihood of adopting a directed transport state when not colocalized with APs (Figure 4E). We also observed one case in which, after a β‐actin mRNA molecule traversed an AP, a small fragment of F‐actin moved along with the β‐actin mRNA molecule in retrograde direction (Figure S3A,B; Movie S8). This observation supports that axonal β‐actin mRNP complexes interact with APs (Figure S3C). Collectively, our results show that β‐actin mRNA travels through the axon shaft and stochastically docks at APs, which are potential sites of branch formation.

## 3 DISCUSSION

Observation of mRNA molecules in live neurons has allowed us to understand the process of mRNA localization in neuronal subcompartments. Previous studies have provided valuable information about the localization and translation of mRNA in axons using nonspecifically labeled mRNA or reporter constructs containing the 3′‐UTR of the mRNA of interest.^6^, ^15^ However, most of the previous methods are limited in that they cannot be used to observe the localization process of a particular mRNA species. Using the MS2 system to visualize single species of mRNA,^19^ we previously investigated β‐actin mRNA transport dynamics in dendrites.^14^ Here, by combining it with a microfluidic system, we studied the localization process of endogenous β‐actin mRNA in axons in real‐time. We found that axonal β‐actin mRNAs exhibited less directed motion and slower speed than dendritic β‐actin mRNAs in mature hippocampal neurons. Though the movement of β‐actin mRNA was generally subdiffusive in axons, some mRNA molecules underwent superdiffusive transport in the axon shaft. Moreover, smFISH analysis showed that axonal β‐actin mRNAs were localized in F‐actin‐rich areas, and real‐time imaging revealed that axonal β‐actin mRNAs docked at APs, which are precursors of axonal filopodia or branches.^27^ Taken together, our findings suggest that axonal β‐actin mRNA undergoes subdiffusive movement caused by APs and that localization of β‐actin mRNA to an AP supplies the protein for a branch to grow from the AP, helping to develop axonal arbors.

Interestingly, we found that axonal β‐actin mRNAs showed less directed movement than dendritic β‐actin mRNAs in mature hippocampal neurons. Though axons are much longer than dendrites, the less directed movement of axonal β‐actin mRNA may imply that a significant fraction of the mRNA is permanently localized rather than repeatedly cycling through the neurite as in the “sushi‐belt model” of mRNA transport in dendrites.^28^ Moreover, axonal β‐actin mRNA mostly showed confined diffusion but had a higher local diffusion coefficient than dendritic β‐actin mRNA, possibly because of the myosin‐induced movement^9^ in the AP. The observation of distinct β‐actin mRNA movement in axons and dendrites reflects the differences in the cytoskeletal configuration,^29^ the variety of motor protein combinations,^30^ different *trans*‐acting factors^31^, ^32^, ^33^ such as adenomatous polyposis coli,^34^, ^35^ and the distribution of late endosomes and lysosomes that are reported to carry RNA granules.^36^, ^37^, ^38^ The mobility of mRNA could also be affected by the translation status. It has been shown that the diffusion coefficient of mRNA increases after treatment with a translation inhibitor in developing axons^5^ and embryonic fibroblasts.^39^ Translation imaging techniques using SunTag system^40^ have also been used to investigate the mobility of mRNA undergoing translation in dendrites.^41^, ^42^ Wang et al.^41^ and Wu et al.^42^ reported that dendritic mRNAs showed directed transport while undergoing active translation. It would be also interesting to investigate the translational status of axonal mRNAs using a similar method.

While our experiments were done in mature neurons, there are numerous reports of mRNPs moving in a highly processive microtubule‐dependent manner in axons of developing neurons.^31^, ^43^ For example, fast directed transport of β‐actin mRNA over long distances have been observed in developing neurites of chick forebrain neurons,^44^ radial glia basal processes,^45^ and growing axons of retinal ganglion cells (RGCs).^5^ Therefore, the characteristics of axonal mRNA transport may differ by several variables such as developmental stages and cell/culture types.

Our results also showed that the type of motion adopted by β‐actin mRNA differed among the specific axonal subcompartments. A previous study on β‐actin mRNA trafficking in axons using molecular beacon also reported that axonal β‐actin mRNA showed more directed transport in the axon shaft than in the growth cone.^5^ Moreover, several studies revealed anchoring of β‐actin mRNA by actin filaments in developing axons^5^, ^9^ as well as the leading edge of fibroblasts^46^ and neuronal dendrites.^4^ These results are consistent with our observation that β‐actin mRNA docks at APs. Previous reports found that ZBP1, one of the RBPs that bind to β‐actin mRNA, was associated with the actin‐based motor protein myosin‐Va, and that knockdown of myosin‐Va increased the number of motile ZBP1 particles.^9^ Therefore, our observation of β‐actin mRNA traveling with a F‐actin fragment suggests that β‐actin mRNA molecules may interact with APs via myosin‐Va and ZBP1.

APs, which are formed along axons via the PI3K pathway, are precursors of filipodia and branches.^27^ Translation machinery including rRNAs, mRNAs, and mitochondria are found at the branch points,^6^ and the inhibition of de novo β‐actin synthesis impaired the emergence of new branches.^15^ Therefore, the localization of β‐actin mRNA at APs may be required for filopodium or branch formation, supplying the necessary amount of nascent β‐actin proteins to support the development of axonal arbor architecture and generate complex synaptic connections.

## 4 METHODS

### 4.1 Microfluidic device fabrication

Microfluidic devices were fabricated by soft lithography as described previously.^22^ The master with the positive relief features of negative photoresist SU‐8 (MicroChem) was developed on a 4‐in. silicon wafer by photolithography and used to generate a mold from which the device was reproduced. A 10:1 (wt/wt) solution of polydimethylsiloxane (PDMS; Sylgard 184; Dow Corning) and a curing agent were mixed gently and poured on the master. After degassing in a vacuum chamber to remove bubbles for approximately 20 min, the mold was thermally cured on a hot plate to obtain replica molds. The reservoir regions for the cell culture medium were punched out with a biopsy punch (6 mm). After oxygen plasma treatment for 60 s to bond the PDMS mold and cover glass, the device was incubated in a dry oven at 80°C for at least 48 h to maintain hydrophobic conditions. The device was sterilized by ultraviolet irradiation before the experiment.

### 4.2 Primary mouse neuron cultures and transfection

All animal experiments were conducted in accordance with a protocol approved by the Institutional Animal Care and Use Committee (IACUC) at Seoul National University. Primary hippocampal neurons were cultured from 1‐day‐old MCP × MBS mouse pups using a previously described method.^47^ In brief, the hippocampus was excised from the brain of each of three to four pups and dissociated with trypsin. For the culture of neurons on the microfluidic devices, the devices were coated with poly‐d‐lysine (10 mg/ml), incubated for at least 6 h and seeded with 2 × 10^5^ neurons. For culture in glass‐bottom dishes, the dishes were coated with poly‐d‐lysine (2 mg/ml) and seeded with ~10^5^ dissociated neurons. The neuronal cultures were grown in vitro for 8–14 days in Neurobasal‐A medium (Gibco) supplemented with B‐26 (Gibco), Glutamax (Gibco), and Primocin (Invivogen) at 37°C in 5% CO_2_.

### 4.3 Imaging of single mRNA molecules in live neurons

Live‐neuron imaging experiments were performed as described previously.^47^ Prior to imaging, the culture medium was removed from the neuronal cultures and replaced with HEPES‐buffered saline containing 119 mM NaCl, 5 mM KCl, 2 mM CaCl_2_, 2 mM MgCl_2_, 30 mM d‐glucose, and 20 mM HEPES (pH 7.4). Fluorescence images were acquired on an Olympus IX73 inverted microscope with a U Apochromat 150 × 1.45 NA total internal reflection fluorescence (TIRF) objective (Olympus), two iXon Ultra 897 EMCCD cameras (Andor), an MS‐2000 XYZ automated stage (ASI), and a Chamlide TC top‐stage incubator system (Live Cell Instruments). A 488‐nm diode laser (Cobolt) was used to visualize β‐actin mRNA, and the fluorescence emission was filtered with a 525/50 bandpass filter (Chroma). To observe F‐actin in live neurons, a 561‐nm diode laser (Cobolt) was used, and the fluorescence emission was filtered with a 630/75 bandpass filter (Chroma). Because of photobleaching, the time scale of each mRNA‐imaging experiment differed according to the purpose of the experiment. To compare the movement of mRNA molecules in dendrites and axons, time‐lapse images were acquired at 5–20 frames per second (fps) for ~1 min. To image mRNA molecules moving in the axonal subcompartments, time‐lapse images were acquired at 1–0.2 fps for 1–15 min. For dual‐color imaging of F‐actin and β‐actin mRNA molecules, time‐lapse images were acquired at 0.2 fps for 1–20 min.

### 4.4 smFISH

Hippocampal neuron cultures at DIV11 were transfected with CMV‐GFP‐UtrCH (Addgene, #26737) by using Lipofectamine 2000 (Thermo Fisher Scientific). The next day, the neurons were fixed with 4% paraformaldehyde and then washed for 10 min with phosphate‐buffered saline (PBS) supplemented with 1 mM MgCl_2_ (PBSM). The neurons were then permeabilized in PBS containing 0.1% Triton X‐100 for 10 min. After two washes in PBSM, the cultured neurons were preincubated for 10 min in prehybridization buffer containing 50% formamide and 2× SSC in RNase‐free water and then hybridized overnight at 37°C in hybridization buffer containing 2× SSC, 10% formamide, 10% dextran sulfate, bovine serum albumin (20 mg/ml), sheared salmon sperm DNA, *Escherichia coli* tRNA and 50‐mer DNA probes. We designed three Quasar 570 dye (Biosearch)‐conjugated probes to target the MBS linker sequence: (i) LK20 (5′‐TTTCTAGAGTCGACCTGCAG‐3′), (ii) LK51‐1 (5′‐CTAGGCAATTAGGTACCTTAG‐3′), and (iii) LK‐51‐2 (5′‐CTAATGAACCCGGGAATACTG‐3′). The neurons were then washed first with a prehybridization buffer and then with 2× SSC and PBSM. smFISH images were acquired on an Olympus IX73 inverted wide‐field microscope with a U Apochromat 150 × 1.45 NA TIRF objective (Olympus). Two‐channel 16 × 16 grid *z*‐stack imaging (from −10 to 10 μm at intervals of 0.4 μm) was performed to obtain a broad view of axon branches. A 488‐nm diode laser (Cobolt) and 561‐nm diode laser (Cobolt) were used to visualize F‐actin and β‐actin mRNA, respectively. The fluorescence emission signals were filtered with 525/50 and 630/75 bandpass filters and simultaneously detected by two EMCCD cameras. To analyze the β‐actin mRNA in axons, we first stitched the grid images and segmented the axons using custom MATLAB scripts. To detect mRNA FISH products, we used FISH‐quant software. We then manually classified the mRNA molecules according to whether they were colocalized with APs.

### 4.5 Quantification and statistical analysis

To analyze mRNA movement, we cropped the region of each image containing mRNA molecules and straightened it (using the *Straighten* function in Fiji software). We then classified each mRNA molecule according to its subcellular localization (bouton, filopodium or shaft). Because mRNA signals in axons were too dim to track automatically, we used HybTrack, a tracking program that combines manual and automatic tracking approaches.^23^ To assess the mobility of the mRNA molecules, the MSD, $\rho_{n}$, was calculated from the N‐frame‐long mRNA track by using the following formula:

$$\rho_{n}=\rho \left(n∆t\right)=\sum_{i=1}^{N-n}\left({\left(x_{i+n}-x_{i}\right)}^{2}+{\left(y_{i+n}-y_{i}\right)}^{2}\right)/\left(N-n\right),$$

where Δ*t* is the frame interval and *x*_*i*_ and *y*_*i*_ are the coordinates of the mRNA molecule in the *i*th frame. The exponent of the MSD, *β*, was calculated by linear fitting from the log‐scaled MSD. The fitting range differed depending on the length and interval of the mRNA track (for a 5–20 fps image taken over ~1 min, the range was 1–2 s; for a 0.2–1 fps image taken over 1–15 min, the range was 3–9 s; and for a 0.2 fps image taken over 1–20 min, the range was 10–40 s). mRNA tracks acquired at 5–20 fps were used to calculate the diffusion coefficient by dividing the linear fitted slopes of the MSDs at 0.2, 0.3, and 0.4 s by 4. To measure the speed of the mRNA molecules, we measured the run length and run time in the section where the mRNA moved more than 1.5 μm in one direction (anterograde or retrograde).

The HMM‐Bayes software was used to infer the transient motion state of the mRNA molecules.^26^ We ran the analysis with two maximum possible states, predicting the mRNA trajectory as diffusion only, directed transport only, two types of diffusion, diffusion mixed with directed transport, or two types of directed transport. Among the possible states, we adopted the maximum likelihood states. For diffusion that had a diffusion coefficient of less than 0.005 μm^2^/s, we define the state as stationary.

The statistical tests used, the number of samples, and the *p* values are specified in the respective figure legends. In Figure 1G, the two Gaussian distributions composing the speed distribution of axonal β‐actin mRNA were verified using the Bayesian information criterion.

## AUTHOR CONTRIBUTIONS

Byung Hun Lee performed all experiments and data analysis. Byung Hun Lee, Seokyoung Bang, and Seung‐Ryeol Lee designed and fabricated the microfluidic devices. Byung Hun Lee and Hye Yoon Park wrote the manuscript. Noo Li Jeon and Hye Yoon Park conceived and supervised the work.

## CONFLICT OF INTEREST

The authors declare no conflict of interest.

## Supporting information


**FIGURE S1** The difference in mRNA movement in proximal and distal dendrites**. (A)** TAMSD exponents of β‐actin mRNAs located in proximal and distal dendrites (n = 215 proximal dendritic mRNAs, n = 46 distal dendritic mRNAs, *** *P* < 10^−14^ by the two‐sample Kolmogorov–Smirnov test). **(B)** Diffusion coefficients of proximal and distal dendritic β‐actin mRNAs (*** *P* < 10^−19^ by the two‐sample Kolmogorov–Smirnov test).
**FIGURE S2. (A)** Kymograph of the time‐lapse image in the right panel of Figure 2A. Scale bars: (horizontal) 10 μm and (vertical) 5 min. **(B)** Images of a β‐actin mRNA molecule at three time points: 1 min, 4 min and 20 min (yellow, green and blue dotted lines in (A), respectively) after imaging began. Two β‐actin mRNA molecules (red and blue arrows) were localized near a potential filopodium, and a filopodium developed after a few minutes.
**FIGURE S3. (A)** Dual‐color time‐lapse images of β‐actin mRNA (green) and F‐actin (red). A β‐actin mRNA molecule (white arrow) underwent retrograde transport and traversed an AP. After traversing the AP, the β‐actin mRNA molecule was accompanied by an F‐actin fragment. **(B)** Enlarged images of the area enclosed in the blue dotted box in (A). Scale bars, 10 μm (A) and 5 μm (B). **(C)** Hypothetical schematic of β‐actin mRNP. An mRNP particle including ZBP1, β‐actin mRNA, myosin‐Va, and dynein is transported along a microtubule carrying a fragment of F‐actin.

**MOVIE S1** Movement of dendritic β‐actin mRNA. Movie was taken at 20 frames per second showing the distinct rest and run states of the β‐actin mRNA shown in Figure 1C.

**MOVIE S2** Movement of β‐actin mRNA in axon. Movie was taken at 20 frames per second showing the confined diffusive motion of the axonal β‐actin mRNA shown in Figure 1C.

**MOVIE S3** An example of axonal β‐actin mRNA inside the bouton shown in Figure 2A. Movie was taken at 20 frames per second.

**MOVIE S4** An example of axonal β‐actin mRNA near the filopodium shown in Figure 2A. Movie was taken at 20 frames per second.

**MOVIE S5** An axonal β‐actin mRNA moving along the axon shaft shown in Figures 2A and S2. The mRNA changed its motion near the filopodium. Movie was taken every 3 s.

**MOVIE S6** An example of β‐actin mRNA in the actin patch shown in Figure 4A. The axonal β‐actin mRNA (green) showed confined diffusive motion inside the F‐actin (red) rich area. Movie was taken every 3 s.

**MOVIE S7** Axonal β‐actin mRNAs that passed through or anchored to the actin patch shown in Figure 4A. mRNA #1 and #3 showed directed transport without docking to the actin patch (red), whereas mRNA #2 anchored to the actin patch. Movie was taken every 5 s.

**MOVIE S8** Axonal β‐actin mRNA that moved with F‐actin fragment after passing through the actin patch shown in Figure Supplement 3. The mRNA (green) showed retrograde transport and passed through the actin patch (red). After passing the actin patch, the mRNA carried F‐actin fragment. Movie was taken every 5 s.

## Data Availability

All materials generated in this study are available from the lead contact with a completed material transfer agreement.
