# Supplementary material for: Dynamics of axonal β‐actin mRNA in live hippocampal neurons
Source: Traffic. 2022 Aug 31;23(10):496–505. doi: 10.1111/tra.12865 (PMC9804286; doi:10.1111/tra.12865)
Supplement: Supplementary file 1 — FIGURE S1 The difference in mRNA movement in proximal and distal dendrites. (A) TAMSD exponents of β‐actin mRNAs located in proximal and distal dendrites (n = 215 proximal dendritic mRNAs, n = 46 distal dendritic mRNAs, *** P < 10−14 by the two‐sample Kolmogorov–Smirnov test). (B) Diffusion coefficients of proximal and distal dendritic β‐actin mRNAs (*** P < 10−19 by the two‐sample Kolmogorov–Smirnov test). FIGURE S2. (A) Kymograph of the time‐lapse image in the right panel of Figure 2A. Scale bars: (horizontal) 10 μm and (vertical) 5 min. (B) Images of a β‐actin mRNA molecule at three time points: 1 min, 4 min and 20 min (yellow, green and blue dotted lines in (A), respectively) after imaging began. Two β‐actin mRNA molecules (red and blue arrows) were localized near a potential filopodium, and a filopodium developed after a few minutes. FIGURE S3. (A) Dual‐color time‐lapse images of β‐actin mRNA (green) and F‐actin (red). A β‐actin mRNA molecule (white arrow) underwent retrograde transport and traversed an AP. After traversing the AP, the β‐actin mRNA molecule was accompanied by an F‐actin fragment. (B) Enlarged images of the area enclosed in the blue dotted box in (A). Scale bars, 10 μm (A) and 5 μm (B). (C) Hypothetical schematic of β‐actin mRNP. An mRNP particle including ZBP1, β‐actin mRNA, myosin‐Va, and dynein is transported along a microtubule carrying a fragment of F‐actin. [file TRA-23-496-s008.docx]

**Supplementary Figures**


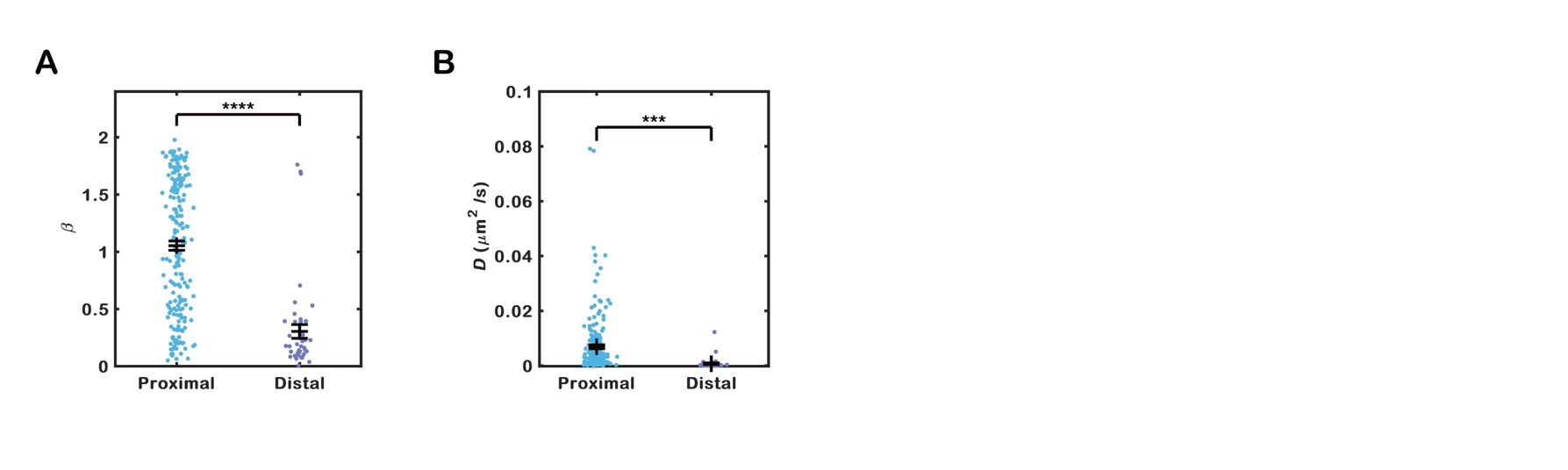


**Figure Supplement 1.** The difference in mRNA movement in proximal and distal dendrites**. (A)** TAMSD exponents of β-actin mRNAs located in proximal and distal dendrites (n = 215 proximal dendritic mRNAs, n = 46 distal dendritic mRNAs, *** *P* < 10^-14^ by the two-sample Kolmogorov-Smirnov test). **(B)** Diffusion coefficients of proximal and distal dendritic β-actin mRNAs (*** *P* < 10^-19^ by the two-sample Kolmogorov-Smirnov test).


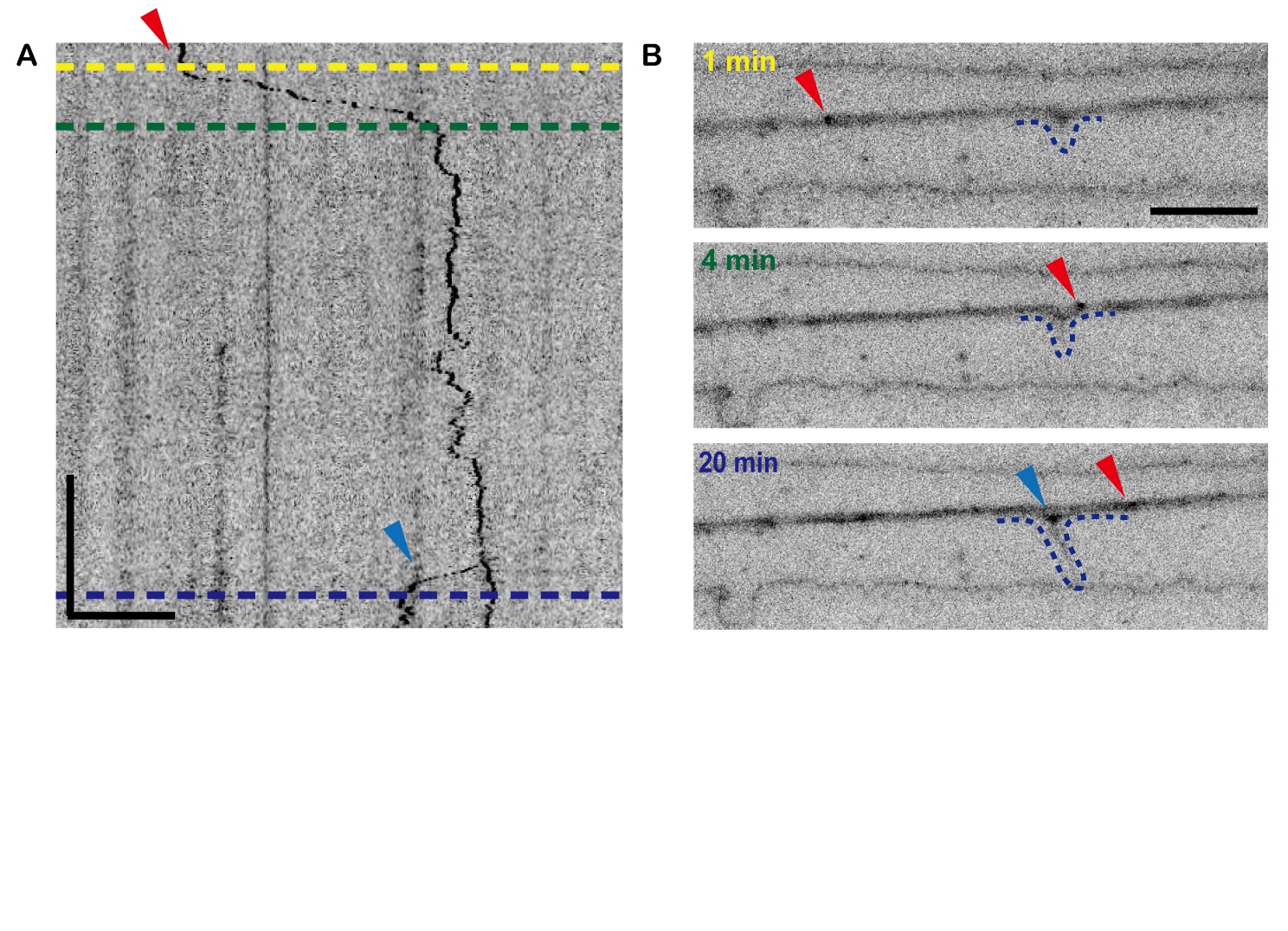


**Figure Supplement 2. (A)** Kymograph of the time-lapse image in the right panel of Figure 2A. Scale bars: (horizontal) 10 µm and (vertical) 5 min. **(B)** Images of a β-actin mRNA molecule at three time points: 1 min, 4 min and 20 min (yellow, green and blue dotted lines in (A), respectively) after imaging began. Two β-actin mRNA molecules (red and blue arrows) were localized near a potential filopodium, and a filopodium developed after a few minutes.


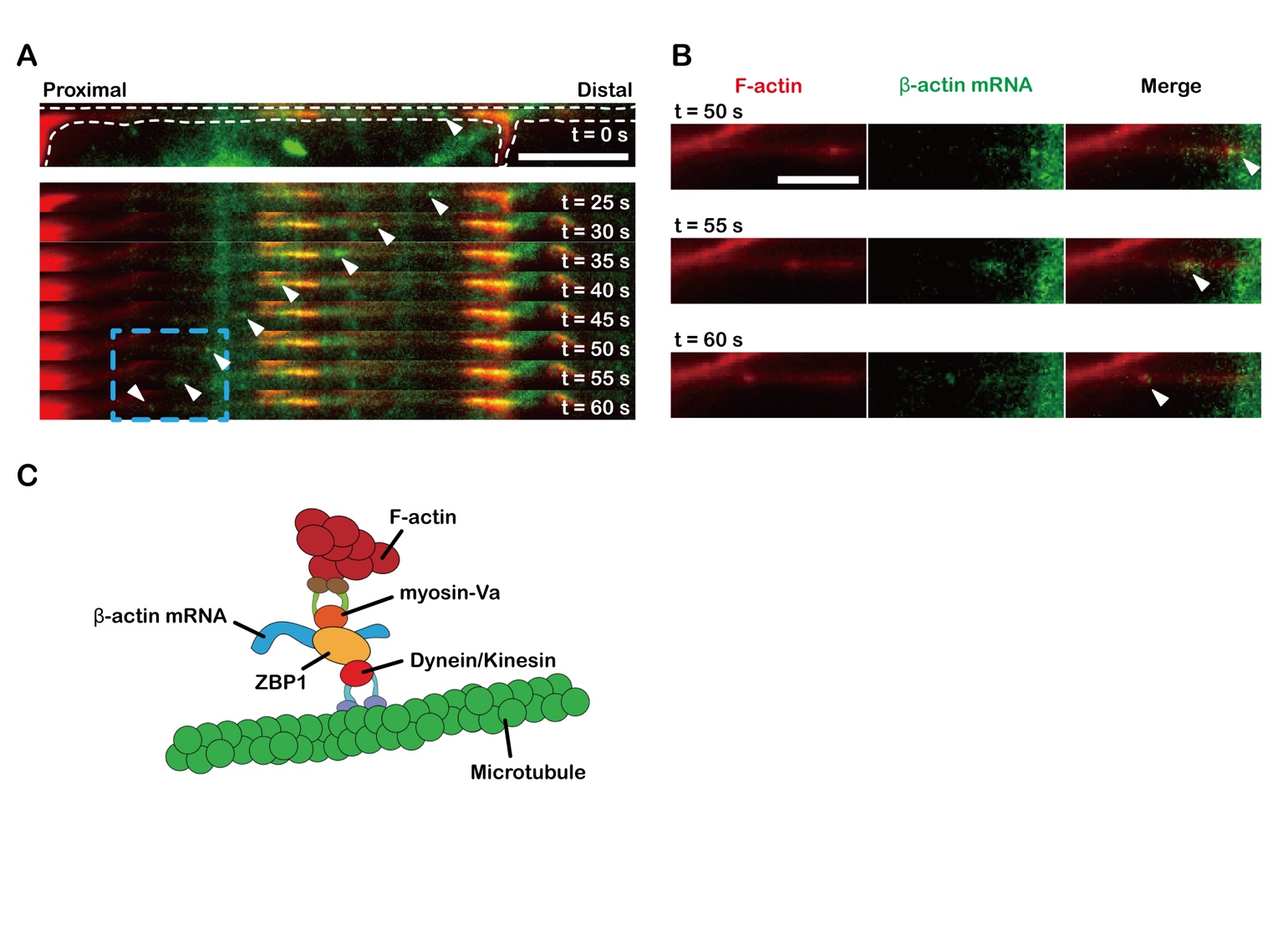


**Figure Supplement 3. (A)** Dual-color time-lapse images of β-actin mRNA (green) and F-actin (red). A β-actin mRNA molecule (white arrow) underwent retrograde transport and traversed an AP. After traversing the AP, the β-actin mRNA molecule was accompanied by an F-actin fragment. **(B)** Enlarged images of the area enclosed in the blue dotted box in (A). Scale bars, 10 μm (A) and 5 μm (B). **(C)** Hypothetical schematic of β-actin mRNP. An mRNP particle including ZBP1, β-actin mRNA, myosin-Va, and dynein is transported along a microtubule carrying a fragment of F-actin.

**Legends for Supplementary Movies S1 to S8**

**Movie S1.** Movement of dendritic β-actin mRNA. Movie was taken at 20 frames per second showing the distinct rest and run states of the β-actin mRNA shown in Figure 1C.

**Movie S2.** Movement of β-actin mRNA in axon. Movie was taken at 20 frames per second showing the confined diffusive motion of the axonal β-actin mRNA shown in Figure 1C.

**Movie S3.** An example of axonal β-actin mRNA inside the bouton shown in Figure 2A. Movie was taken at 20 frames per second.

**Movie S4.** An example of axonal β-actin mRNA near the filopodium shown in Figure 2A. Movie was taken at 20 frames per second.

**Movie S5.** An axonal β-actin mRNA moving along the axon shaft shown in Figure 2A and Figure Supplement 2. The mRNA changed its motion near the filopodium. Movie was taken every 3 seconds.

**Movie S6.** An example of β-actin mRNA in the actin patch shown in Figure 4A. The axonal β-actin mRNA (green) showed confined diffusive motion inside the F-actin (red) rich area. Movie was taken every 3 seconds.

**Movie S7.** Axonal β-actin mRNAs that passed through or anchored to the actin patch shown in Figure 4A. mRNA #1 and #3 showed directed transport without docking to the actin patch (red), whereas mRNA #2 anchored to the actin patch. Movie was taken every 5 seconds.

**Movie S8.** Axonal β-actin mRNA that moved with F-actin fragment after passing through the actin patch shown in Figure Supplement 3. The mRNA (green) showed retrograde transport and passed through the actin patch (red). After passing the actin patch, the mRNA carried F-actin fragment. Movie was taken every 5 seconds.
